# Inadequate Intake of Dietary Fibre in Adolescents, Adults, and Elderlies: Results of Slovenian Representative SI. Menu Study

**DOI:** 10.3390/nu13113826

**Published:** 2021-10-27

**Authors:** Barbara Koroušić Seljak, Eva Valenčič, Hristo Hristov, Maša Hribar, Živa Lavriša, Anita Kušar, Katja Žmitek, Sanja Krušič, Matej Gregorič, Urška Blaznik, Blaž Ferjančič, Jasna Bertoncelj, Mojca Korošec, Igor Pravst

**Affiliations:** 1 Computer Systems Department, Jožef Stefan Institute, SI-1000 Ljubljana, Slovenia; eva.valencic@ijs.si; 2 Jožef Stefan International Postgraduate School, SI-1000 Ljubljana, Slovenia; 3 Priority Research Centre in Physical Activity and Nutrition, Faculty of Health and Medicine, School of Health Sciences, The University of Newcastle, Callaghan, NSW 2308, Australia; 4 Nutrition Institute, Tržaška Cesta 40, SI-1000 Ljubljana, Slovenia; hristo.hristov@nutris.org (H.H.); masa.hribar@nutris.org (M.H.); ziva.lavrisa@nutris.org (Ž.L.); anita.kusar@nutris.org (A.K.); katja.zmitek@vist.si (K.Ž.); sanja.krusic@nutris.org (S.K.); igor.pravst@nutris.org (I.P.); 5 Higher School of Applied Sciences (VIST), Gerbičeva Cesta 51A, SI-1000 Ljubljana, Slovenia; 6 National Institute of Public Health, Trubarjeva 2, SI-1000 Ljubljana, Slovenia; matej.gregoric@nijz.si (M.G.); urska.blaznik@nijz.si (U.B.); 7 Biotechnical Faculty, University of Ljubljana, Jamnikarjeva 101, SI-1000 Ljubljana, Slovenia; blaz.ferjancic@bf.uni-lj.si (B.F.); jasna.bertoncelj@bf.uni-lj.si (J.B.); mojca.korosec@bf.uni-lj.si (M.K.)

**Keywords:** dietary fibre, dietary fibre intake, 24 h recall, EU Menu, Slovenian population

## Abstract

Dietary fibre has proven to promote healthy body mass and reduce the risk of non-communicable diseases. To date, in Slovenia, there were only a few outdated studies of dietary fibre intake; therefore, we explored the dietary fibre intake using food consumption data collected in the SI.Menu project. Following the EU Menu methodology, data were collected on representative samples of adolescents, adults, and elderlies using a general questionnaire, a food propensity questionnaire, and two 24 h recalls. The results indicate that the intake of dietary fibre in Slovenia is lower than recommended. The proportion of the population with inadequate fibre intakes (<30 g/day) was 90.6% in adolescents, 89.6% in adults, and 83.9% in elderlies, while mean daily fibre intakes were 19.5, 20.9, and 22.4 g, respectively. Significant determinants for inadequate dietary fibre intake were sex in adolescents and adults, and body mass index in adults. The main food groups contributing to dietary fibre intake were bread and other grain products, vegetables and fruits, with significant differences between population groups. Contribution of fruits and vegetables to mean daily dietary fibre intake was highest in elderlies (11.6 g), followed by adults (10.6 g) and adolescents (8.5 g). Public health strategies, such as food reformulation, promoting whole-meal alternatives, consuming whole foods of plant origin, and careful planning of school meals could beneficially contribute to the overall dietary fibre intake in the population.

## 1. Introduction

Though the importance of dietary fibre in human health is well established, knowledge of dietary fibre intake in some countries is still lacking. In Slovenia, fairly recently, a national food consumption survey was carried out which enabled estimation of dietary fibre intake among all the populations. Food consumption data collected in such a way are valuable; however, their matching with compositional data on dietary fibre is a challenging task because dietary fibre refers to a number of compounds with different chemical structures for which compositional data for a complete set of foods are still missing.

The Codex Alimentarius [1] and the European Food Safety Authority (EFSA) [2] define dietary fibre as a compound consisting of at least 10 monomeric units which are not hydrolysed in the human small intestine and are passing to the large intestine. Oligosaccharides with 3 to 9 monomeric units are also considered as dietary fibre [2]. Some types of dietary fibre can be metabolised by the gut microbiota and can form energy-yielding substances [3]. Therefore, the energy value of 2 kcal per gram of dietary fibre is considered in the calculation of the energy value of foods [4].

Dietary fibre has been recognised as a very important part of healthy diets. By increasing stool frequency and bulk volume, dietary fibre helps with defecation and might protect us against heart diseases, hypertension, and certain types of cancer [5,6]. Several recent sources also reveal the importance of dietary fibre intake in local colon microbiota [7,8,9]. Colonic bacteria produce enzymes that can partly degrade dietary fibre molecules through the process of fermentation. By-products of the dietary fibre fermentation (i.e., short-chain fatty acids and gases) are very important as they influence the composition of the gut microbiota, leading to a healthier gut microbiome which helps to regulate the immune system [10].

The terms soluble and insoluble dietary fibre are sometimes used to describe the types of dietary fibre in our diet. In the review of Stephen et al. [11], a difference in lowering risk factors for cardiovascular diseases CVD based on the solubility of dietary fibre was reported, in which soluble dietary fibre shows a greater effect. A similar result was confirmed for the reduction in appetite in weight management—soluble dietary fibre shows better appetite suppression. Despite some exceptions, the division of dietary fibre based on its solubility is the closest approximation for predicting the fermentability of dietary fibre. The majority of soluble dietary fibre is fermentable, and the fermentation of dietary fibre in the colon is an important process for colon health influencing the human organism as a whole [12]. However, very few food composition databases (FCDBs) include information about the content of (in)soluble dietary fibre. National FCDBs also lack complete data on other types of dietary fibre, such as non-starch polysaccharides (NPS; cellulose, hemicelluloses, pectins, hydrocolloids (i.e., gums, mucilages, β-glucans), and fructans); resistant (short-chain) oligosaccharides (fructooligosaccharides (FOS), galactooligosaccharides (GOS), and other resistant oligosaccharides); resistant starch (physically enclosed starch, some types of raw starch granules, retrograded amylose, and chemically and/or physically modified starches); lignin (aromatic polymer associated with the dietary fibre polysaccharides). These types of dietary fibre have various physicochemical properties, which are of key importance for their physiological effects.

Recommendations about adequate dietary fibre intake are not harmonised. Based on the available evidence on bowel function, the EFSA’s NDA Panel concluded that dietary fibre intake of 25 g per day is adequate for normal laxation in adults, while in children, from the age of one year, they recommended 2 g of dietary fibre per MJ [2]. Considering the evidence of benefit to health associated with consumption of diets rich in dietary fibre-containing foods, the NDA Panel also noted that dietary fibre intake of greater than 25 g per day can reduce risks of coronary heart disease and type 2 diabetes, and improve body mass maintenance. In Slovenia, D-A-CH recommendations (D-A-CH present country identification for the countries Germany (D), Austria (A), and Switzerland (CH), for which these guidelines were developed) have been adopted by the Slovenian Ministry of Health since 2004 [13]. This guidance generally recommends an intake of 30 g of dietary fibre per day [14].

Daily dietary fibre requirements can be met by consuming plenty of fresh fruits and vegetables, potatoes, whole-grain cereals, legumes, and pulses, nuts, and seeds. The EFSA’s NDA Panel estimated that the average dietary fibre intakes in Europe vary from 10 to 20 g per day in young children (of age less than 10 to 12 years), from 15 to 30 g per day in adolescents, and from 16 to 29 g per day in adults [2]. However, no recent data on the intake of dietary fibre are available for Slovenia. The only nationally representative study on adults was conducted back in 1995/96, indicating a mean dietary fibre daily intake of 20.1 g (20.3 in men and 19.9 in women) [15,16]. Additionally, almost two decades ago, Fidler Mis et al. conducted a nationally representative study among adolescents and reported a dietary fibre intake of 28 ± 9 g among boys and 31 ± 11 g among girls [17].

The main objective of the study presented in this paper was to estimate intake of dietary fibre exploiting the most recent data, collected within the national food consumption survey (SI.Menu 2017/2018). Our focus was on adolescents, adults, and elderly populations. Due to the lack of studies on the estimation of dietary fibre intake that differentiate between different types of fibre, which have different physiological roles in the human body, we also aimed to evaluate a proportion of the (in)soluble fibre in people’s diets. Secondary objectives were to investigate the prevalence of inadequate dietary fibre intakes and associations with different socio-demographic and lifestyle determinants and to identify major sources of dietary fibre in different age groups.

## 2. Material and Methods

### 2.1. Study Design and Subjects

The study was conducted as part of a national cross-sectional food consumption survey, named SI.Menu, which was aimed at collecting food consumption data in Slovenia in the period from March 2017 to April 2018. The survey was designed considering the EFSA Guidance on European Union Menu Methodology [18]. Details about the methodology and sample characteristics are published elsewhere [19,20]. We should note that Slovenia has a population of only 2.06 million people (2007 census data). The participants were stratified into three age groups: adolescents (10–17 years old), adults (18–64 years old), and the elderly (65–74 years old). Sampling was carried out for individuals, which could not be substituted with another household member. A total of 2280 subjects were selected using the Central Register of Population (CRP) of Slovenia according to age, sex, and place of residency (to cover all NUTS-3 statistical regions). In Slovenia, CPR is administered by the Ministry of the Interior of the Republic of Slovenia and represents the central repository and processing of data concerning residents of the Republic of Slovenia; data were provided from the Statistical Office of the Republic of Slovenia. Individuals living abroad, and institutionalised, ill, or people with disabilities were excluded from the study. The selected residents were visited by survey interviewers, who checked the eligibility of the respondents and collected required information by interviews. The interviewers underwent a course in nutrition surveys and nutritional surveillance in practice. In case of any questions or problems during an interview, they had a possibility to contact the project leaders. The survey was completed by a total of 62% of the invited participants (*n* = 1319); the lowest participation rate was observed in adults (57%), while the rate was higher in adolescents (69%) and elderlies (65%). In these two groups, participation rates were very similar between males and females, while in adults, the participation rate of males was notably lower (50 vs. 61%). The study protocol was approved by the National Medical Ethics Committee (KME 53/07/16; approval No. 0120-337/2016 issued on 19.7.2016). Prior to inclusion in the study, all subjects were informed about the study and signed an informed consent form. In the case of adolescents, informed consent was also obtained from the parent or legal guardian.

### 2.2. Food Consumption Data

In the survey SI.Menu, two questionnaires were prepared to enable the collection of food consumption data and corresponding metadata. Additionally, food consumption data were collected with two 24 h dietary recalls:

1. General Questionnaire (GQ) enabled the collection of data for accessing general socio-demographic, socio-economic, and lifestyle determinants, such as place of living, number of household members, marital status, level of education, monthly net income of the household, dietary and consumer habits, as well as usual frequency and duration of physical activity.
2. Food Propensity Questionnaire (FPQ) was used to record the usual frequency of consumption of specific foods in the last 12 months: Altogether, 78 food items were allocated into 9 food groups. For example, within cereals and cereal products, FPQ included separate questions for white bread, whole-grain bread, etc. The FPQ considered the following frequency response options: *never, 1–3 times per month or less, once per week, 2–3 times per week, 4–6 times per week, 1–2 times per day or more*. More details about the SI.Menu FPQ can be found elsewhere [19].
3. Information about the participant’s dietary habits was collected by two non-consecutive 24 h dietary recalls which were carried out up to three weeks apart (71% of the recalls were performed on workdays and 29% on weekends). The majority (87%) of the second 24 h recalls was collected within 7 days; the rest was completed within the next two weeks. The first 24 h recalls occurred at the participant’s home and were completed by the participant’s interviewer. The second 24 h recall was performed over the telephone or at participants’ homes (in cases when they could not be reached over the telephone). The latter was only performed for a small sample of participants. Portion sizes were estimated with the help of a nationally adjusted picture book, containing 46 pictures of different food products or simple recipes presented in 6 different portion sizes [19]. For home-cooked (mixed) dishes, participants were asked to provide recipes. When this was not available, and for all outdoor dining, standard recipes from the Open Platform for Clinical Nutrition (OPEN) [21] were used.

At the first interviewer’s visit, the general questionnaire and the FPQ were completed by the interviewer. The interviewer also collected anthropometric data, measured the participant’s body height (m) and body mass (kg) using a portable, calibrated scale. These data were used to determine the body mass index (BMI; kg/m^2^) considering the overweightness cut-off point at 25 kg/m^2^, except for adolescents, where sex/age-adjusted cut-off points (>1SD) were applied [22,23]. Additionally, using the provided information about the usual frequency and duration of physical activity, the participant’s International Physical Activity Questionnaire (IPAQ) score [24] was calculated. In the GQ, participants were asked to select a type of residential area (city/town; suburbs; village type areas), and this information was used to assign them into urban, intermediate, and rural areas, respectively.

Food consumption data collected by the 24 h recalls were analysed using a food composition database created as part of the Open Platform for Clinical Nutrition (OPEN) [21]. The OPEN database includes compositional data on generic foods and some branded foods and provides a list of ingredients for traditional and other recipes frequently consumed in Slovenia. For foods not covered in national compositional data, the OPEN database uses European (EuroFIR) and United States Department of Agriculture (USDA) food composition databases [25].

### 2.3. Assessment of Dietary Fibre Content

Data on dietary fibre intake were extracted from the SI.Menu food consumption dataset. The SI.Menu dataset contains both simple foods and complex foods and recipes. In some cases, the ingredient information and recipe were provided by the subjects, while in other cases, traditional or commonly used recipes (pre-collected in OPEN) were used. For these complex foods and recipes, a disaggregation method was applied considering both the yield and retention factors [26], to calculate their dietary fibre contents. Foods extracted from the 24 h recalls were inserted into OPEN and checked by a nutrition expert. In case of missing information on the amount of dietary fibre in specific food products, missing data were supplemented with the data from the Fineli database [27]. This is the Finish National Food Composition Database, maintained by the National Institute for Health and Welfare, which also contains information about soluble and insoluble dietary fibre. If a product was not found in this database, the Danish [28] and German [29] databases were also used. Altogether, 67.9% of food items were determined to be sources of total dietary fibre, and for 60.0% of those food items, we were able to estimate the amount of (in)soluble dietary fibre, corresponding to 77.3%, 79.3%, and 81.7% in the total fibres intake for adolescents, adults, and elderly populations, respectively. For the remaining part of the dietary fibre, we were unable to distinguish between soluble and insoluble fractions. Missing data on the content of (in)soluble dietary fibre were observed in spices, nuts, and seeds, dried vegetables and fruits, processed meat, processed fish, ready-to-eat foods, baby foods, cakes, muffins and pastry, sauces, side dishes, food supplements, and meal replacements. Each food was allotted into one of the 101 food categories, of which 78 were included in FPQ.

For the purpose of identifying major sources of dietary fibre, foods were additionally categorised into categories developed within the Global Food Monitoring Initiative (GFMI) [30], adapted for use in Slovenia [20], as in previously published SI.Menu studies [20]. The following major food categories were included: fruit and vegetables; bread and bakery products; cereal and cereal products; convenience foods; snack foods; other.

### 2.4. Study Sample

Details about the exclusion criteria are previously explained [20]. Under- and overreporting were assessed using the cut-off points method initially described by Goldberg et al. [31] and further adapted by Black et al. [32]. The method is based on the ratio of reported daily energy intake and basic metabolic rate (BMR). BMR was calculated based on sex, age, body height, and body weight using the method described by Harris et al. [33] and adapted by Roza and Shizgal [34]. The calculated cut-off points for 24 h recalls for under- and overreporting were 0.41 and 2.46, respectively. Participants reporting energy intakes of less than 500 kcal were also excluded from the analyses. After exclusion of 97 subjects (incomplete or missing anthropometric data: *n* = 12; missing one of the 24 h recall data: *n* = 36; under/overreporting: *n* = 49), a study sample included 1248 subjects—468 adolescents, 364 adults, and 416 elderlies. Exclusion of subjects with missing anthropometric data was conducted because body height and weight were also needed to determine BMR, while subjects with only one 24 h recall were excluded because dietary fibre intakes were estimated with the use of the multiple source method [35], which only works with at least two 24 h recalls.

### 2.5. Data Analyses

Usual intake distributions per age group adjusted for within individual day-to-day variation were modelled with the multiple source method (MSM) [35], using both 24 h recalls and PFQ data. The method is characterised by a two-part shrinkage technique applied to residuals of two regression models—one for the daily food intake data and one for consumption frequency (FPQ). The shrunken residuals are back-transformed to their original scale, and the individual usual intake is obtained by multiplication of the frequency and amounts result. The MSM was used to correct dietary data for intra- and inter-personal variabilities. Assessment of dietary fibre intake was performed only for food categories which contained food items as sources of fibre. Age group, sex, and body mass index (BMI) were considered as covariates. After the MSM was applied, the usual daily intake of dietary fibre was calculated for each individual. The same approach was also applied separately for soluble and insoluble dietary fibre. The share of insoluble dietary fibre in the total dietary fibre content (calculated as the sum of soluble and insoluble dietary fibre) was further calculated for each individual. Similarly, daily energy intake was calculated for each subject and used for the calculation of total dietary fibre in grams per daily energy intake of 1000 Kcal.

Descriptive characteristics (mean, median, and proportions) are presented for age cohorts. Prevalence for inadequate daily intake of total dietary fibre was calculated using two cut-off values: 30 g (nationally adapted D-A-CH recommendation [13,14]) and 25 g (EFSA’s guidance [2]). To account for the differences in participation rates in different population groups, population weighing was used in the presentation of these epidemiological results. Weighting was performed using iterative proportional fitting [36] for deviances in age and sex, with consideration of 2017 census data.

Logistic regression analysis was used to investigate associations between the prevalence of inadequate dietary fibre intakes with different socio-demographic and lifestyle determinants, separately for each population group. For this analysis, inadequate dietary fibre intake was determined using a cut-off value of 30 g/day, using individual usual dietary fibre intakes, estimated by MSM [35]. The following parameters were used in the models: sex, place of living, BMI, and IPAQ levels for all age groups, education, income for adults and elderly, and employment status for adults only. Logistic regression model parameters were estimated by the maximum likelihood method; odds ratios (ORs) with 95% confidence intervals (CIs) were calculated. All regression analyses were conducted on samples with excluded missing values (family net income: *n* = 57 (adults) and 40 (elderly); IPAQ: *n* = 5 (adolescents), 4 (adults), 6 (elderly)).

The relative contribution of different food categories to usual daily dietary fibre intake was calculated using previously described GFMI food categories. Separately for each age group, we summed contributions to daily fibre intake in all food groups and calculated their percentage (%) in the total dietary fibre intake in the age group. Further, we calculated mean usual dietary fibre intakes for three food categories, which were identified as major contributors to fibre intake (fruits and vegetables, bread and bakery products, cereal and cereal products). For each subject, food category contribution to daily fibre intake (in grams per day) was used for the calculation of age group mean and SD. To evaluate the differences in contributions of these food categories in the daily fibre intakes between age groups, we used one-way ANOVA, followed by Levene’s test for homogeneity of variance. Multiple-comparison post hock test with Bonferroni correction was used in cases with equal variances; alternatively, Games–Howell adjustment was used.

All statistical analyses were performed using STATA version 15.1 (StataCorp LLC, College Station, TX, USA), while individual dietary intakes were estimated using the online tool MSM V1.0.1 (https://msm.dife.de/ (assessed on 26 October 2021); the Department of Epidemiology of the German Institute of Human Nutrition Potsdam-Rehbrücke, Germany). Differences were considered as significant at *p* < 0.05.

## 3. Results

Characteristics of the study sample are presented in Table 1. Sex ratios in male and female adolescents, adults, and the elderly were close to 1:1. Most subjects in all age groups lived in rural places of living (approx. 55%), while subjects living in urban places were in minority (slightly more than 25%). The remaining participants lived in intermediate places of living. Almost 70% of adults and more than 80% of the elderly had no university degree. The largest proportion of adults was employed (62.1%), 11.5% of subjects were unemployed, 8.8% were students, and 17.6% were retired. Body mass index (BMI) was estimated as normal in the majority of adolescents (64.3%), while in adults and the elderly, the percentages of overweight and obese subjects were dominant (59.3% of adults and 74% of the elderly).

Population-weighting was used to account for different participation rates in different population groups. Population weighted mean intake of total dietary fibre was 19.5, 20.9, and 22.4 g/day for adolescents, adults, and elderly populations, respectively (Table 2). The distribution of the mean usual intake of total dietary fibre in the age cohorts is presented in Supplementary Figure S1. With consideration of energy intake, women had a higher mean intake of dietary fibre in all three age groups (11.9, 13.7, and 14.4 g per 1000 kcal, respectively), while their actual mean daily dietary fibre intake was somewhat lower than in men.

Next, we examined a proportion of the population with insufficient dietary fibre intake, with consideration of nationally adapted D-A-CH recommended intake of at least 30 g of total dietary fibre daily (Table 2). While 90.6% of adolescents and 89.6% of adults had insufficient dietary fibre intake, the percentage of the elderly who daily consume less than 30 g/day of total dietary fibre was 83.9%. The trend was similar if the cut-off point was set to 25 g dietary fibre per day, which is an amount recommended by the EFSA to prevent constipation. The proportion of the population consuming less than 25 g/day of total dietary fibre was again the lowest among the elderly (70.8%), while among adults and adolescents, these proportions accounted for 75.5% and 83.0%. We also estimated the proportion of insoluble dietary fibre in people’s diets. This proportion was estimated with consideration of all foods, for which data about the content of (in)soluble dietary fibre was available. Insoluble dietary fibres prevailed in all three age groups with small differences in the mean share of insoluble dietary fibre (63.9, 64.9, and 65.2, respectively; Table 2).

Multivariable logistic regression analyses were used to investigate associations of the prevalence of inadequate dietary fibre intakes (<30 g per day) with different socio-demographic and lifestyle determinants. Due to the study sampling approach, which provided representative samples separately for adolescents, adults, and elderlies, regression analysis was carried out separately for each population group. Most of the socio-economic and lifestyle variables were used in all the modes, except for education and income (only used in adults and elderlies) and employment (only used for adults). Results are presented in Table 3. It should be noted that in contrast to results in Table 2, herein, reported prevalence of insufficient dietary fibre intake is provided for the study sample, without population weighting. Sex was identified as a notable predictor of dietary fibre intake. In women, odd ratios for inadequate dietary fibre were significantly higher in adolescents (OR 2.00; 95%CI 1.05, 3.81) and adults (OR 2.78; 95%CI 1.21, 6.38). A contrary trend was observed in elderlies (OR 0.68, 95%CI: 0.36–1.29) but without a statistically significant difference (*p* = 0.237). In adults, overweight/obesity was found strong and significant predictor for lower OR (2.86; 95%CI 1.24, 6.59) for meeting recommended dietary fibre intake. Without statistical difference, a similar trend was observed in adolescents but not in elderlies. Surprisingly, higher family net income was found close to significant (*p* < 0.1) predictor for insufficient dietary fibre intake (OR 2.07, 95%CI: 0.85, 5.05) in adults but not in elderlies.

**Figure 1:**
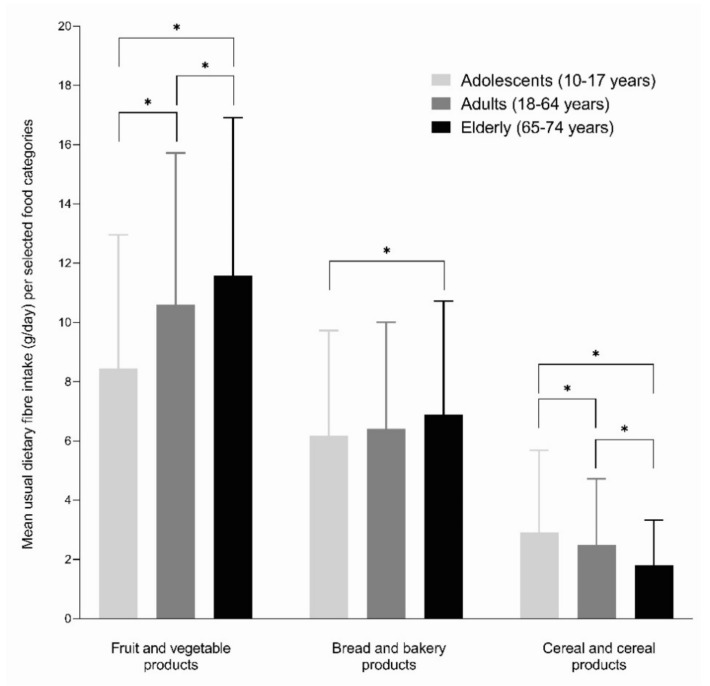
Mean usual dietary fibre intakes (g/day) from selected food categories among different age groups. The symbol * denotes a trend in the difference.

Since dietary fibre includes heterogeneous components found in different foods (mostly of plant origin), we explored collected data to figure out which food categories mostly contributed to the dietary fibre intake among different age groups. First, we calculated the relative contribution of different food categories to the total consumption of dietary fibre, separately for all three age groups (Supplementary Table S1). Fruits and vegetables, bread and bakery products, and cereal products were found as the main contributors to the total dietary fibre intake, and therefore subject to further analyses, enabling statistical comparison between age groups. Mean usual dietary fibre intakes from these three food categories are presented in Figure 1. Significant age-dependent trend was observed in fruit and vegetable products, which contributed 8.5 g, 10.6 g, and 11.6 g of dietary fibre in adolescents, adults, and elderlies, respectively. Somewhat similar trends were observed for bread and bakery products, where the difference was only significant between adolescents and elderlies (6.2 g vs. 6.9 g). However, a contrary trend with significant differences was observed in cereals and cereal products, which contributed to 2.9 g, 2.5 g, and 1.8 g of dietary fibre in adolescents, adults, and elderlies, respectively. We should note that in all three age groups subjects consumed more dietary fibre from vegetables (mostly fresh, and including mushrooms, legumes, pulses, and sprouts) than from fruits. The diversity of foods in the category of vegetables was high and included all types of foods usually consumed in the Mediterranean diet and also traditional products for our region. For example, fermented vegetables (such as sauerkraut and fermented turnip) are regularly consumed in Slovenia, especially in wintertime.

## 4. Discussion

Reported results indicate that the mean usual intake of total dietary fibre among the residents in Slovenia is notably lower than the national recommendations (30 g/day, adopted D-A-CH guidelines). Highest dietary fibre intake was observed in the elderly population (mean 22.4 g/day; 13.2 g/1000 kcal = 3.2 g/MJ), followed by adults (20.9 g; 12.2 g/1000 kcal = 2.9 g/MJ) and adolescents (19.5 g/day; 11.2 g/1000 kcal = 2.7 g/MJ). In comparison, a previous study reported a slightly lower mean dietary fibre daily intake of 20.1 g in Slovenian adults in 1995/96 [15,16]. In adolescents, in the period 2003–2005, Fidler Mis et al. also reported a very similar intake of dietary fibre (2.6 and 2.8 g/MJ in boys and girls, respectively) [17]. However, it should be noted, that the latter study was conducted using a very different methodology (food frequency questionnaires without food recalls), and their reported intake of dietary fibre in grams per day is somewhat higher (28 and 31 g in boys and girls, respectively).

Regarding the proportion of insoluble dietary fibre, the results of the presented study showed that the largest proportion could be observed in the elderly (65.2%; 14.6 g/day), followed by adults (64.9%; 13.6 g/day) and adolescents (63.9%; 12.5 g). Although there is no dietary reference intake for soluble or insoluble fibre, many experts recommend about one-fourth (25%) of total dietary fibre intake—6 to 8 g per day—should come from soluble fibre, and the remaining 75% should come from insoluble fibre. Considering this recommendation, we can conclude that the consumption of soluble fibre (from foods such as oats, brussels sprouts, beans, peas, apples, oranges, nuts, and flax, and other seeds) is well satisfied by all the Slovenian populations (7.8 g, 7.3 g, and 7 g are daily consumed by the elderly, adults and adolescents, respectively). It seems that the more significant problem is with the consumption of insoluble fibre. No Slovenian population satisfies the daily dietary reference intake of 19 to 22 g of insoluble fibre (from foods such as wheat bran; vegetables such as green beans and dark leafy greens; root vegetables such as carrots, beets, and radish; fruit skins; and intact whole grains). However, with this interpretation, we need to emphasise that for about 20% of the total dietary fibre intake (23%, 21%, and 18% for adolescents, adults, and elderly population, respectively), data about the content of (in)soluble fibre were not available. This issue was addressed with a methodological approach—the proportion of insoluble fibre was not calculated as a percentage of total dietary fibre but as a percentage of the sum of soluble and insoluble fibre. This was possible under the assumption that the distribution of (in)soluble fibre in the diet from foods with missing data is comparable with the dietary contribution of foods, for which data were available.

Alarmingly, the proportion of participants with insufficient daily fibre intake (<30 g) was over 80% in all age groups. The results of our study are quite consistent with studies in other countries, which also reported inadequate dietary fibre intake [2,37,38,39,40]. The proportion of the population with insufficient dietary fibre intake was lowest in elderlies (especially females). This could be because they are more likely to suffer from chronic diseases than other populations and are, therefore, more concerned about their nutrition and some of them also follow specific diets [41]. This population also prepares more meals at home from raw ingredients, such as fruits, vegetables, and grains, which also contain more dietary fibre [41].

An interesting finding of our study is that in comparison with men, adolescent and adult women were more likely to have insufficient dietary fibre intake, while the contrary was observed in elderlies. Conversely, in the Irish elderly population [39], women consumed less dietary fibre than men, but their intake of dietary fibre per energy intake was higher than in men, which was also observed in our study. We should also mention a Dutch study on adults (19–69 years) [42], which only reported dietary fibre per energy intake and also observed higher fibre intakes in women. Nevertheless, the majority of the population is far from meeting the recommendations for daily dietary fibre intake, regardless of gender, which is in line with previous reports from Slovenia [43] and elsewhere [39,44].

As mentioned, according to the opinion of the EFSA’s NDA Panel, daily intake of 25 g of dietary fibre is adequate for normal laxation [2], but this lower threshold value is also not met by 83.0% of adolescents, 75.5% of adults, and 70.8% of the elderly population. We should also mention that the European Society for Paediatric Gastroenterology Hepatology and Nutrition (ESPGHAN) also recommends that during adolescence the dietary fibre intake should be gradually increased to reach a dietary fibre intake of 30 g/day [45], which is far from the current status in Slovenia. Results of our study highlighted that adolescents should be encouraged to swipe white bread for wholemeal varieties, while comparison with other age groups (Figure 1) also indicates the high potential of vegetables and fruits to increase intake of dietary fibre. For example, in adolescents, the contribution of fruits and vegetables to daily dietary fibre intake is 3.1 g lower than in elderlies. At the same time, adolescents consume approximately twice more amount of fibre from white bread than from brown bread. On the other hand, other cereal products were more important sources of dietary fibre for adolescents than for adults and elderlies. Other studies also highlighted that adolescents more commonly consume snacks (usually high energy foods), and lack main meals (most commonly breakfast) or have irregular meals, and have low consumption of fruits and vegetables [46]. To improve intake of dietary fibre, consuming more of other wholesome, fibre-rich foods should be supported in adolescents, as well as in other populations.

In a UK study [40], an investigation of the associations between dietary fibre intakes from different food sources with measures of body composition was performed. Comparison of our results with this study showed that in both countries, the percentage of the adult population which meets the recommended intake for total dietary fibre of more than 30 g per day is about 10% (9% in the UK and 10.4% in Slovenia). In both studies, the main total dietary fibre contributions come from grains, fruits, and vegetables. Vegetables and fruits are good sources of non-starch polysaccharides (NSP) (hemicelluloses and pectin), resistant oligosaccharides, and resistant starch. On the other hand, nuts and seeds—sources of cellulose, hemicelluloses, and also resistant starch [47]—were also notable sources of dietary fibre in Slovenian adults (4.1%), while this was not the case in the UK study (0.7%). This could be explained by the fact that nuts and seeds are popular snacks in Slovenia. However, such snacks are often high in added fat and salt; therefore, healthy alternatives should be encouraged. In both studies, it appeared that the main sources of total dietary fibre from the category of grains are white bread and other grain sources, which are not necessarily whole grain. The adolescents could benefit from the reformulation of breakfast cereals to contain more dietary fibre, as this food group is more popular in this population group than in adults or elderlies. The UK study observed that higher whole-grain and non-whole-grain cereal dietary fibre intakes are associated with lower BMI. However, dietary fibre from whole-grain sources (but not from non-whole-grain sources) was associated with lower waist circumference and percentage of body fat, suggesting that some benefits of dietary fibre from whole grains might also be attributable to other nutrients and non-nutrient components (e.g., phytochemicals) in whole grains. Several other studies also associated insufficient dietary fibre intake with obesity, but results are not always conclusive [38,48,49]. Whole grains include bran as a source of NSPs, and they provide lignin, resistant oligosaccharides, and resistant starch. The SI.Menu data showed that Slovenian residents consume a very wide variety of different whole-grain cereals, including wheat, corn, buckwheat, millet, etc.

In 2017, Stephen et al. published a comprehensive review of dietary fibre intake in European countries, considering data from nearly 140,000 individuals covering a broad age range from early childhood to the elderly [10]. Overall, daily dietary fibre intake for adults living in European countries was estimated to be 18–24 g for men and 16–20 g for women, which is comparable with our results (21.1 g for men and 20.7 g for women). This review highlighted bread and grain products as the largest source of total dietary fibre. Additionally, in our study, a combination of bread, bakery products, and other cereal products were a major source of dietary fibre (45.0%, 40.0%, and 39.0% for adolescents, adults, and elderly, respectively), followed by vegetables and fruits (Supplementary Table S1).

In order to improve the situation in Slovenia, promoting whole-meal options and encouraging food reformulations to increase dietary fibre content could result in higher overall dietary fibre intake in all population groups. Large meta-analyses covering over 1.7 million subjects [50] showed that with every 10 g increase in dietary fibre intake per day, the risk of premature death was lowered by 11%. Considering this, the target recommendation of daily intake of 30 g dietary fibre is very relevant, but we are still far from this target. Our study showed that the dietary fibre intake is very comparable with the situation two decades ago, despite the fact that we have observed notable changes in people’s behaviours [51], and also in the food supply. These changes could result in a lower intake of dietary fibre. Nowadays, food stores are packed with tens of thousands of highly processed foods [52], which became major food sources in different population groups. It is becoming clear that this trend will not be turned around, making reformulation policies even more important. Furthermore, in adolescents, dietary fibre intake could be also improved by careful planning of school meals to include more whole foods of plant origin and refined cereal alternatives, such as whole-meal bread and cereals.

The strength of the study lies in that it was built on the comprehensive nationally representative dataset, collected using internationally harmonised European Union Menu Methodology for food consumption surveys. This cross-sectional study included randomised selection of residents aged 10–74 years. To enable the provision of insights into more vulnerable population groups, sampling was performed separately for adolescents, adults, and the elderly population. Another strength is that data were collected over 12 months to cover all calendar seasons, with quota sampling for each quarter of the year. This means that nationally representative sampling was also achieved during seasons. Furthermore, estimated usual intake was estimated in both 24 h recalls and food propensity questionnaire data. Some limitations should be also mentioned. While we did our best to increase the participation rate, 38% of participants did not respond to our invitation. However, the observed participation rate is common in national dietary studies with randomised sampling, because participation in the survey is voluntary. We also did not use any financial compensations, which could, on one the one hand, increase participation rate, but on the other hand, such an approach would have different effects on different economic groups. However, after a successful face-to-face visit, subjects received a small gift (glass water bottle, bib, headphones, umbrella, or ice bags), as a gratuity gesture and to stimulate participation in a second 24 h dietary recall. Subjects were able to select between these incentives, which were very well accepted. Participation was also affected by season. A lower participation rate was observed during summer when people are come commonly out of home (holidays), and it was highest during the winter season. We should, however, mention that interviewer had to achieve five contacts with the selected subjects. If none were successful, the subject was coded as a non-respondent. We did not use within a household or other substitutions, which would increase the participation rate. In the case of soft refusals, the subject was contacted one more time by a different interviewer. An important limitation of the study is also that national food composition tables did not enable us to estimate fibre content in all of the reported foods. Additional food composition databases were, therefore, used. As already mentioned, the estimation of the proportion of the insoluble dietary fibre (as % of total fibre) was carried out only with consideration of foods for which we were able to estimate the amount of soluble and insoluble dietary fibre. Another limitation is that we did not include the consumption of food supplements with dietary fibre (e.g., *Plantago psyllium*, some prebiotics, mushrooms, algae), which can also present an important source of dietary fibre. We should mention that the penetration of the consumption of such supplements in Slovenia is low. About 10% of Slovenian adults reported at least monthly consumption of probiotic and prebiotic formulations, and 7% are using herbal formulations and plant extracts, while the use of algae and mushrooms formulations is about 2% [41].

## 5. Conclusions

In Slovenia, as in most other European countries, the mean daily intake of the total dietary fibre is much lower than recommended. Sex and BMI were found as the strongest determinants for insufficient daily dietary fibre intake in specific population groups. The main food groups contributing to dietary fibre intake in all population groups were fruits and vegetables, bread, and cereal products. While for the elderly population, there are no previous data for comparison, dietary fibre intake in adolescents and the adult population seems comparable with results of studies, conducted about two decades ago. However, major methodological differences between the studies limit these comparisons. The results of this study indicate that more efficient approaches are needed to increase dietary fibre intake and to meet recommendations, which should exceed food reformulation activities. Considerably higher intake of dietary fibre could be achieved by encouraging consumers for whole-meal options in bread and cereal products and with increased overall consumption of plant-based foods.

## Figures and Tables

**Table 1:** Demographic and lifestyle characteristics of the study sample.

		Age Cohorts		
		Adolescents	Adults	Elderly
		(10–17 Years)	(18–64 Years)	(65–74 Years)
		*n* = 468	*n* = 364	*n* = 416
Age; years—mean (SD)		13.4 (2.37)	43.6 (13.81)	68.7 (2.7)
Place of living—*n* (%)	Rural	270 (57.7)	202 (55.5)	229 (55.1)
	Intermediate	76 (16.2)	56 (15.4)	71 (17.1)
	Urban	122 (26.1)	106 (29.1)	116 (27.9)
Sex—*n* (%)	Male	238 (50.9)	173 (47.5)	213 (51.2)
	Female	230 (49.1)	191 (52.5)	203 (48.8)
Education—*n* (%)	No university degree	n.a.	249 (68.4)	342 (82.2)
	University degree	n.a.	115 (31.6)	74 (17.8)
Family monthly net income—*n* (%)	Below average	n.a.	118 (38.4)	269 (71.5)
	Above average	n.a.	189 (61.6)	107 (28.5)
BMI—mean (SD)		21.0 (4.2)	26.7 (5.2)	28.4 (5.0)
*n* (%)	Normal	301 (64.6)	148 (40.7)	108 (26.0)
	Overweight and obese	167 (35.7)	216 (59.3)	308 (74.0)
IPAQ—*n* (%)	Low intensity	108 (23.3)	127 (35.3)	137 (33.4)
	Moderate	141 (30.5)	108 (30.0)	133 (32.4)
	High intensity	214 (46.2)	125 (34.7)	140 (34.2)
Employment status—*n* (%)	Employed	n.a.	226 (62.1)	n.a.
	Unemployed	n.a.	42 (11.5)	n.a.
	Student	n.a.	32 (8.8)	n.a.
	Retired	n.a.	64 (17.6)	n.a.

**Notes**: Body mass index (BMI) was considered to be normal when it was below 25 kg/m^2^, except for adolescents, where sex/age-adjusted cut-off points [22,23] were used; International Physical Activity Questionnaire (IPAQ); standard deviation (SD); not applicable (n.a). Table adapted from [20].

**Table 2:** Population-weighted usual daily dietary fibre intake and proportion of the population with inadequate daily fibre intakes.

	Adolescents (10–17)			Adults (18–64)			Elderly (65–74)		
	All	Male	Female	All	Male	Female	All	Male	Female
Weighted * N (%)	150,674 (78.2)	75,580 (50.2)	73,094 (49.8)	1,302,132 (78.2)	670,464 (51.5)	631,668 (48.5)	212,793 (12.8)	100,247.5 (47.1)	112,545.5 (52.9)
Sample N (%)	468 (100)	238 (50.9)	230 (49.2)	364 (100)	173 (47.5)	191(52.5)	416 (100)	213 (51.2)	203 (48.8)
Intake of total dietary fibre									
Mean (95%CI) [g/day]	19.5 (18.8–20.2)	20.5 (19.6–21.5)	18.3 (17.3–19.3)	20.9 (20.1–21.7)	21.1 (19.9–22.3)	20.7 (19.6–21.8)	22.4 (20.5–24.3)	20.9 (19.6–22.1)	23.9 (20.7–27.0)
Median [g/day]	18.8	19.6	17.5	19.7	20.1	19.2	20.6	18.8	21.8
Mean (95%CI) [g per 1000 Kcal/day] **	11.2 (10.8–11.7)	10.6 (10.0–11.9)	11.9 (11.3–12.5)	12.2 (11.7–12.7)	10.7 (10.1–11.3)	13.7 (13.0–14.3)	13.2 (12.3–14.0)	11.8 (10.8–12.8)	14.4 (13.5–15.3)
Prevalence for inadequate daily intake of total dietary fibre ***									
									
<25 g/day	83.0 (78.4–86.7)	79.1 (71.6–85.0)	87.2 (81.6–91.3	75.5 (69.9–80.3)	74.2 (66.1–80.9)	76.8 (68.7–83.4)	70.8 (61.5–78.7)	77.6 (67.6–85.2)	64.6 (51.2–76.1)
<30 g/day	90.6 (87.1–93.1)	88.1 (82.4–92.2)	93.2 (88.9–95.9)	89.6 (85.6–92.6)	88.6 (82.5–92.7)	90.7 (84.7–94.6)	83.9 (74.0–90.5)	91.0 (85.1–94.7)	77.4 (61.0–88.2)
Share of insoluble dietary fibre intake as % of total daily fibre intake ****									
Mean (95%CI)	63.9 (63.3–64.5)	63.9 (63.0–64.7)	63.9 (63.1–64.6)	64.9 (64.4–65.4)	64.9 (64.1–65.7)	64.9 (64.2–65.5)	65.2 (63.9–66.4)	64.6 (62.3–66.9)	65.7 (65.0–66.4)
Median	63.9	63.7	64.2	65.3	65.4	65.3	65.3	64.6	65.6

Notes: * Number of citizens and respective share in the population in terms of age and sex cohorts (census data in 2017). ** Conversion factor into g/MJ is 0.239; *** Prevalence for inadequate daily intake of total dietary fibre was calculated using two cut-off vales: 30 g (nationally adapted D-A-CH recommendation) [13,14], and 25 g (EFSA’s guidance [2]); **** Based on the available data for content of (in)soluble dietary fibre in foods (corresponding to 77.3%, 79.3% and 81.7% in the total fibres’ intake for adolescents, adults, and elderly population, respectively).

**Table 3:** Association between prevalence of inadequate daily intake of dietary fibre (<30 g/day) and sex, place of living, education, family net income, BMI, IPAQ, employment for diferent age groups.

Variable		Adolescents (10–17 Years old)			Adults (18–64 Years old)			Elderly (65–74 Years old)		
		Prevalence (%)	Crude OR	Adjusted OR	Prevalence (%)	Crude OR	Adjusted OR	Prevalence (%)	Crude OR	Adjusted OR
Overall		422 (90.2)			329 (90.4)			367 (88.2)		
Sex	Male	208 (87.4)	1	1	152 (87.9)	1	1	191 (89.7)	1	1
	Female	214 (93.0)	1.93 (0.98–3.90)	2.00 (1.05–3.81)	177 (92.7)	1.75 (0.81–3.85)	2.78 (1.21–6.38)	176 (86.7)	0.75 (0.39–1.43)	0.68 (0.36–1.29)
Place of living	Village	240 (88.9)	1	1	184 (91.1)	1	1	200 (87.3)	1	1
	Town	70 (92.1)	1.46 (0.57–4.46)	1.45 (0.57–3.67)	51 (91.1)	1.00 (0.34–3.61)	0.68 (0.22–2.09)	65 (91.6)	1.57 (0.60–4.83)	1.89 (0.74–4.89)
	City	112 (91.8)	1.40 (0.64–3.23)	1.35 (0.63–2.89)	94 (88.7)	0.77 (0.33–1.82)	0.80 (0.33–1.90)	102 (87.9)	1.06 (0.51–2.26)	1.34 (0.64–2.82)
Education	No university degree		n.a.	n.a.	228 (91.6)	1	1	306 (89.5)	1	1
	University degree				101 (87.8)	0.66 (0.31–1.48)	0.76 (0.31–1.74)	61 (82.4)	0.55 (0.27–1.21)	0.48 (0.22–1.04)
Family net income	Below average		n.a.	n.a.	105 (89.0)	1	1	237 (88.1)	1	1
	Above average				170 (90.0)	1.10 (0.48–2.48)	2.07 (0.85–5.05)	91 (85.1)	0.77 (0.39–1.58)	0.87 (0.43–1.76)
BMI	Normal	267 (88.7)	1	1	130 (87.8)	1	1	99 (91.7)	1	1
	Overweight and obese	155 (92.8)	1.64 (0.80–3.59)	1.75 (0.87–3.51)	199 (92.1)	1.62 (0.76–3.48)	2.86 (1.24–6.59)	268 (87.0)	0.61 (0.25–1.33)	0.61 (0.27–1.35)
IPAQ	Low intensity	95 (88.0)	1	1	113 (89.0)	1	1	118 (86.1)	1	1
	Moderate	127 (90.1)	1.24 (0.51–2.99)	1.14 (0.50–2.58)	96 (88.9)	0.99 (0.40–2.47)	0.96 (0.38–2.45)	119 (89.5)	1.36 (0.62–3.10)	1.53 (0.72–3.25)
	High intensity	195 (91.1)	1.40 (0.61–3.14)	1.39 (0.65–2.97)	117 (93.6)	1.81 (0.68–5.18)	2.00 (0.72–5.58)	124 (88.6)	1.25 (0.58–2.73)	1.33 (0.63–2.79)
Employment	Employed		n.a.	n.a.	198 (87.6)	1	1		n.a.	n.a.
	Unemployed				39 (92.9)	1.84 (0.53–9.89)	3.49 (0.71–17.10)			
	Student				30 (93.8)	2.12 (0.49–19.25)	1.81 (0.37–8.94)			
	Retired				62 (98.9)	4.38 (1.05–38.89)	5.52 (1.13–27.07)			

Notes: Confidence interval (CI); Body mass index (BMI) was considered as normal below 25 kg/m^2^, except for adolescents, where gender/age adjusted cut-off points [22,23] were used. Logistic regression analysis conducted on samples with excluded missing values (family net income: *n* = 57 (adults) and 40 (elderly); IPAQ (International Physical Activity Questionnaire): *n* = 5 (adolescents), 4 (adults), 6 (elderly)). Cut-off odds ratios calculated with threshold of 30 g dietary fibre daily; Following parameters were found significant: *p* < 0.05 sex (adolescents); *p* < 0.05 sex (adults), *p* < 0.1 income (adults), *p* < 0.05 BMI (adults); *p* < 0.1 education (elderly).

## Data Availability

The data presented in this study are available on request from the corresponding author.

## Supplementary Materials

The following are available online at www.mdpi.com/article/10.3390/nu13113826/s1, Supplementary Figure S1: Histogram of distribution of daily dietary fibre intakes for different age groups (adolescents: age 10–17 years; adults: age 18–64 years; elderly: age 65–74 years), Supplementary Table S1: Relative contribution of selected food categories to usual daily dietary fibre intake among different age groups (% of total dietary fibre intake).
