# Supplementary material for: Inadequate Intake of Dietary Fibre in Adolescents, Adults, and Elderlies: Results of Slovenian Representative SI. Menu Study
Source: Nutrients. 2021 Oct 27;13(11):3826. doi: 10.3390/nu13113826 (PMC8619009; doi:10.3390/nu13113826)
Supplement: Supplementary file 1 [file nutrients-13-03826-s001.zip › nutrients-1409934-supplementary.pdf]

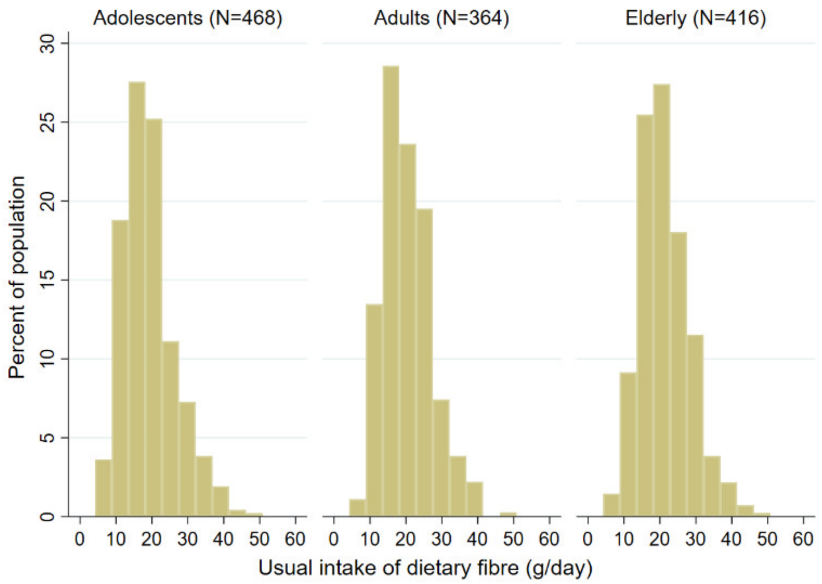

Supplementary Figure S1. Histogram of distribution of daily dietary fibre intakes for different age groups (Adolescents: age 10-17 years; Adults: age 18-64 years; Elderly: age 65-74 years).

Supplementary Table S1. Relative contribution of selected food categories to usual daily dietary fibre intake among different age groups (% of total dietary fibre intake).

Food categories	Food category contribution in total dietary fibre daily diet (%)		
	**Adolescents (10-17)**	**Adults (18-64)**	**Elderly (65-74)**
**Fruit and vegetables**	**41.94**	**49.66**	**52.57**
Vegetables	24.74	28.60	30.45
Fresh vegetables	*22.22*	25.22	*27.19*
Canned vegetables	*2.52*	3.37	*3.27*
Fruit	14.58	16.56	18.98
Fresh fruit	*13.54*	15.11	*17.05*
Canned fruit	*1.04*	1.45	*1.92*
Dried fruit	*0.72*	0.99	*1.03*
Nuts and seeds	2.20	4.13	2.56
Jam and spreads	0.42	0.38	0.58
**Bread and bakery products**	**31.00**	**29.73**	**31.37**
Bread	24.26	25.31	27.47
White bread	*16.13*	14.35	*13.56*
Brown bread	*8.13*	10.96	*13.91*
Biscuits	2.74	1.87	1.49
Cakes, muffins and pastry	4.01	2.55	2.41
**Cereal and cereal products**	**14.01**	**10.71**	**7.66**
Breakfast cereals & unprocessed cereals	8.26	5.93	4.38
Cereal bars	*0.61*	0.25	*0.01*
Pasta	4.46	4.10	2.82
Rice	0.68	0.43	0.46
**Convenience foods**	**3.25**	**2.64**	**2.43**
**Snack foods**	**2.68**	**1.26**	**0.38**
**Other**	**11.74**	**10.04**	**9.39**
